# A transcriptomic atlas at bulk and single-cell levels identifies novel transcriptional and splicing regulators of ECM homeostasis in osteoarthritis

**DOI:** 10.3389/fgene.2025.1690319

**Published:** 2025-12-16

**Authors:** Xiguang Ye, Chunyan Zheng, Hao Li

**Affiliations:** 1 Hubei University of Chinese Medicine, Wuhan, Hubei, China; 2 Affiliated Hospital of Hubei University of Chinese Medicine, Hubei Provincial Hospital of Traditional Chinese Medicine, Hubei Provincial Institute of Traditional Chinese Medicine, Wuhan, Hubei, China; 3 The First Clinical Medical School, Hubei University of Chinese Medicine, Wuhan, Hubei, China

**Keywords:** alternative splicing, extracellular matrix, osteoarthritis, RNA-binding protein, transcription factor

## Abstract

Osteoarthritis (OA) is a common chronic degenerative joint disease. Chondrocytes undergo dynamic changes during the pathogenesis of OA, and the destruction of the extracellular matrix (ECM) and its homeostatic disruption are hallmarks of OA. This study explores the roles of transcriptional and alternative splicing (AS) mechanisms in regulating extracellular matrix (ECM) homeostasis in osteoarthritis (OA), using bulk and single-cell RNA-sequencing data. By analyzing two OA transcriptome datasets, we identified differentially expressed genes (DEGs) that are enriched in ECM-related pathways and constructed a regulatory network between differentially expressed transcription factors (DE TFs) and ECM-related DEGs. This revealed the potential roles of transcription factors ELF3 and DDIT3 in regulating the expression of *COL3A1*, *COL5A2*, and *S100A4*. Single-cell RNA-sequencing data further validated the expression patterns of *ELF3* and *DDIT3* in distinct chondrocyte subtypes. Additionally, by analyzing AS events, we identified the RNA-binding protein (RBP) KHDRBS3 as a regulator of AS for the ECM-related gene *IL16*. Aberrant changes in these events may impact the ECM environment of chondrocytes and contribute to the pathogenesis of OA. This study, for the first time, dissects the regulatory models in OA cartilage at both transcriptional and post-transcriptional levels. These findings provide novel potential targets for early diagnosis and intervention strategies in OA.

## 1 Introduction

Osteoarthritis (OA) is a common chronic degenerative joint disease and a leading cause of increased global disability and morbidity (Glyn-Jones et al., 2015). The pathogenesis of OA includes the degradation of cartilage matrix, inflammation, fibrosis, failure of cartilage repair, bone remodeling, and aging (Jiang, 2022; Vincent et al., 2022; Li et al., 2023a). During the development of OA, chondrocytes are not static but undergo continuous and complex dynamic changes (Singh et al., 2019). The extracellular matrix (ECM) is a major component of articular cartilage and is crucial for the structural maintenance, metabolic regulation, inflammatory response, and repair processes of the joint (Choi et al., 2023; Chatterjee et al., 2022; Hodgkinson et al., 2022; Gao et al., 2014). The destruction of the chondrocyte ECM and the disruption of its homeostasis are hallmarks of OA (Rahmati et al., 2017; Peng et al., 2021). Transcription factors (TFs), as a class of key proteins that regulate gene expression, can also participate in the pathological process of OA by influencing chondrocyte development and the homeostasis of the ECM (Lefebvre and Dvir-Ginzberg, 2017; Neefjes et al., 2020). However, the dynamic changes of the ECM during the pathogenesis of OA, as well as the intricate regulatory networks underlying these changes, remain to be further elucidated.

Alternative splicing (AS) is the process by which pre-messenger RNA (pre-mRNA) in eukaryotes is spliced at different splice sites to generate a variety of mature mRNA isoforms (Ule and Blencowe, 2019). Numerous studies have illustrated that abnormal AS events are implicated in a wide range of human diseases (Marasco and Kornblihtt, 2023). Recent studies have demonstrated that AS in OA is often associated with genes related to the ECM and collagen (Cai et al., 2019; Zhang et al., 2024a; Liu et al., 2024). However, few studies have systematically explored the role of abnormal AS in the development and progression of OA.

Our study demonstrates that the homeostasis imbalance of the ECM in OA is dually regulated by TFs and AS, with both mechanisms driving cartilage degeneration through complex regulatory networks. The transcription factors ELF3 and DDIT3 regulate the expression of *COL3A1*, *COL5A2*, and *S100A4*. The RNA-binding protein (RBP) KHDRBS3 modulates the AS events of *IL16*. Aberrant changes in these events may impact the ECM environment of chondrocytes and contribute to the pathogenesis of OA. These findings can provide new potential targets for the early diagnosis and intervention strategies of OA.

## 2 Materials and methods

### 2.1 Retrieval and process of public data

**FIGURE 1:**
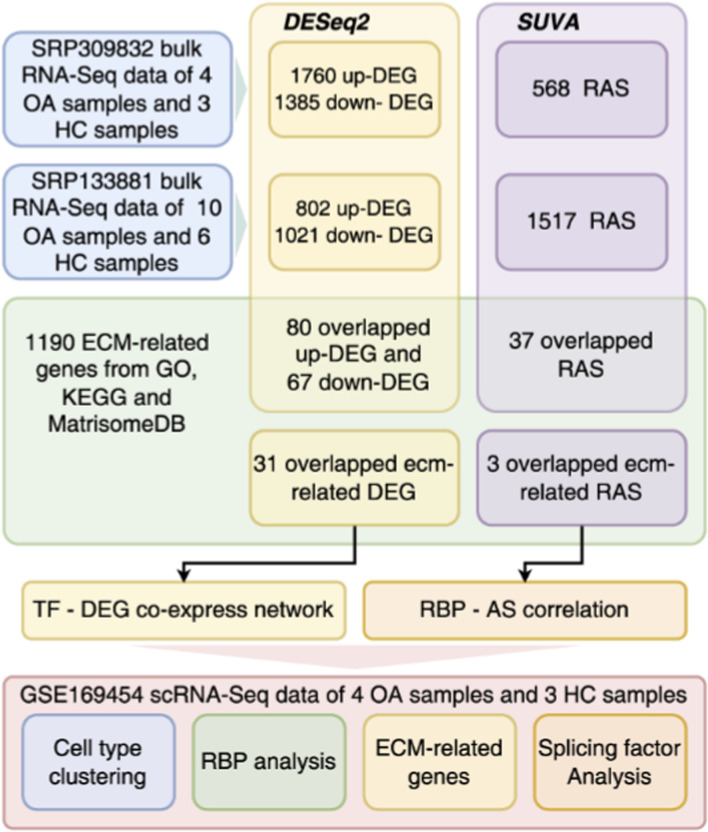
Workflow of bioinformatic analyses in this study. Schematic workflow integrating bulk RNA-seq (GSE168505, GSE111357) and scRNA-seq (GSE169454) analyses.

The overall procedures of our study are illustrated in the flow chart (Figure 1). Public sequence data files of 3 RNA-seq dataset from GSE168505 (SRP309832), GSE111357 (SRP133881) and GSE169454 were downloaded from the Sequence Read Archive (SRA), where GSE168505 and GSE169454 contain cartilage tissues from 4 OA patients and 3 healthy individuals, and GSE111357 includes hip cartilage tissues from 10 OA patients and 6 control subjects (with neck of femur fractures but no OA). SRA Run files were converted to fastq format with NCBI SRA Tool fastq-dump. The raw reads were trimmed of low-quality bases using a FASTX-Toolkit (v.0.0.13; http://hannonlab.cshl.edu/fastx_toolkit/). Then the clean reads were evaluated using FastQC (http://www.bioinformatics.babraham.ac.uk/projects/fastqc).

### 2.2 AS analysis using SUVA

The alternative splicing (AS) events and regulated alternative splicing events (RAS) between healthy controls (HC) and OA samples were defined and quantified using the alternative splicing analysis software SUVA. SUVA is a bioinformatic tool specialized in AS analysis from RNA-seq data, which detects AS events by quantifying splice site usage variation: it first defines events based on annotated genomes and RNA-seq read alignments, then calculates two key metrics—splicing ratio difference (to reflect the magnitude of splicing variation between groups) and reads proportion of SUVA AS event (pSAR, to ensure the reliability of detected events)—to filter high-confidence AS signals; it classifies AS events into five main types (Cheng et al., 2021):

- Alternative 5′splice site (alt5p/A5SS):Shares a common 3′splice site but uses alternative 5′splice sites, leading to transcripts with variable 5′exon lengths.
- Alternative 3′splice site (alt3p/A3SS):Shares a common 5′splice site but uses alternative 3′splice sites, resulting in transcripts with variable 3′exon lengths.
- Intron retention (IR):A full intron is retained in the mature transcript, corresponding to a splice site pair that is either used or unused, leading to transcripts with retained intronic sequences.
- Contain:One splice junction (SJ) is completely contained within another SJ, meaning the sequence of one SJ is a subset of another SJ’s sequence.
- Overlap (olp):Two SJs have distinct splice sites but their junction sequences partially overlap, creating complex splicing patterns with shared sequence regions.

### 2.3 Co-expression analysis of differentially expressed TFs and ECM-related differential expression of genes

The software DEseq2 (Love et al., 2014), which is specifically used to analyze the differential expression of genes, was applied to screen the raw count data for differentially expressed genes (DEGs). The results were analyzed based on the fold change (FC ≥ 2 or ≤0.5) and false discovery rate (FDR≤0.05) to determine whether a gene was differentially expressed. 1190 ECM-related genes were retrieved from the GO (https://www.geneontology.org/), KEGG (https://www.genome.jp/kegg/) and MatrisomeDB (https://matrisomedb.org/).

### 2.4 Prediction of TF motifs and TF binding sites on genes

This study systematically identified TF binding motifs using the JASPAR database (Rauluseviciute et al., 2023). The FIMO module (Grant et al., 2011) from the MEME suite was implemented for genome-wide prediction of TF binding sites, with promoter regions defined as sequences spanning from −1,000 bp upstream to +100 bp downstream relative to the transcription start site (TSS).

### 2.5 Functional enrichment analysis

To identify functional categories of genes, we employed the clusterProfiler package (v4.6.2) (Wu et al., 2021), which enabled us to determine Gene Ontology (GO) terms and KEGG pathways.

### 2.6 Co-expression analysis of splicing ratio of ECM-related RAS and expression of differentially expressed RBPs

Then expression profile of differentially expressed RBPs (DE RBPs) were filtered out from all DEGs according to a catalogue of 2,141 RBPs of human was retrieved from previous report (Castello et al., 2012; Castello et al., 2016; Gerstberger et al., 2014; Hentze et al., 2018). The regulated AS events located in ECM-related genes (ECM-RAS) were screened and analyzed. The co-disturbed network between the expression of DE RBPs and the splicing ratio of ECM-RAS was constructed, with Spearman’s pvalue ≤ 0.05 being retained.

### 2.7 Other statistical analysis

Principal component analysis (PCA) analysis was performed by R package factoextra (https://cloud.r-project.org/package=factoextra) to show the clustering of samples with the first two components. After normalizing the reads by TPM (Tags Per Million) of each gene in samples, in house-script (sogen) was used for visualization of next-generation sequence data and genomic annotations. The pheatmap package (https://cran.r-project.org/web/packages/pheatmap/index.html) in R was used to perform the clustering based on Euclidean distance.

## 3 Results

### 3.1 Analysis of DEGs in OA and control samples

**FIGURE 2:**
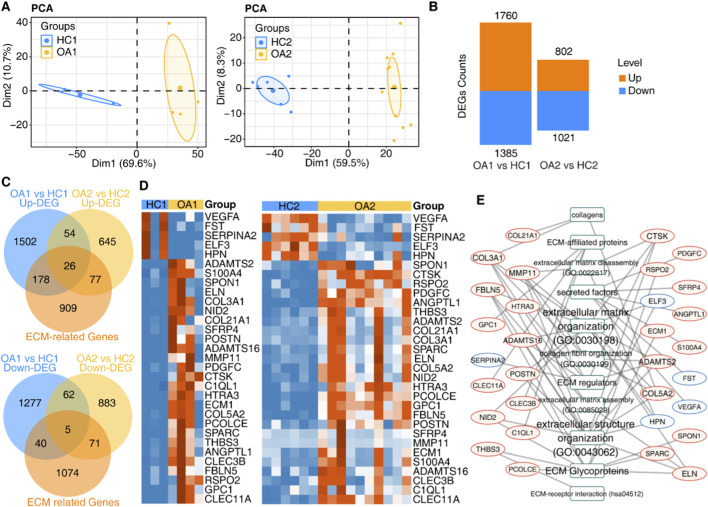
Identification of DEGs and ECM-related DEGs. **(A)** PCA of all expressed genes showing clear separation between OA (red) and HC (blue) groups along PC1. **(B)** Bar plot displaying numbers of up- (orange) and downregulated (blue) DEGs in two datasets. **(C)** Venn diagram illustrating overlap between DEGs and ECM-related genes (n = 31). **(D)** Heatmap of 31 overlapped ECM-related DEGs expression profiles across samples. **(E)** Network showing sources of overlapped ECM-related DEGs, with red/blue borders indicating up/downregulation.

PCA was performed based on the expression levels of all detected genes to explore the global expression pattern. The PCA clustering outcomes indicated that the OA and control groups were distinctly separated along the first principal component (Figure 2A), suggesting significant differences in their global gene expression pattern. We then screened the data for DEGs, which revealed 1760 upregulated genes and 1,385 downregulated genes in Dataset 1 (GSE168505), and 802 upregulated genes and 1,021 downregulated genes in Dataset 2 (GSE111357) (Figure 2B). Gene ontology biological process (GO-BP) analysis indicated enriched terms in which the DEGs were involved. The results showed that in Dataset 1, the upregulated DEGs were mainly related to chemotaxis and cell adhesion, while the downregulated DEGs were primarily associated with mRNA splicing and limb morphogenesis (Supplementary Figure S1A,B). In Dataset 2, the biological processes in the upregulated DEGs mainly involved extracellular matrix organization and extracellular structure organization, while those in the downregulated DEGs were linked to response to biotic stimulus, regulation of inflammatory response, and cell adhesion (Supplementary Figure S1C,D). These findings suggest that the aforementioned biological pathways may play important roles in the pathogenesis of OA.

Considering that the expression changes of ECM-related genes may serve as a hallmark of OA, we further performed an overlap analysis between the 147 overlapping DEGs identified in the two RNA-seq datasets and a list of 1,190 known ECM-related genes. A total of 31 ECM-related DEGs were obtained, with 26 genes commonly upregulated and 5 genes commonly downregulated in both datasets (Figure 2C). The detailed list of genes is provided in Supplementary Table S1, and the changes in gene expression levels are depicted in the heatmap (Figure 2D). The biological processes involved in the 31 ECM-related DEGs mainly include ECM organization, extracellular structure organization, ECM glycoproteins, ECM regulators, and collagen fibril organization (Figure 2E). It can be inferred that these genes are primarily associated with the composition and regulation of the ECM.

### 3.2 Construction of the regulatory network of differentially expressed TFs-ECM-related genes in OA

**FIGURE 3:**
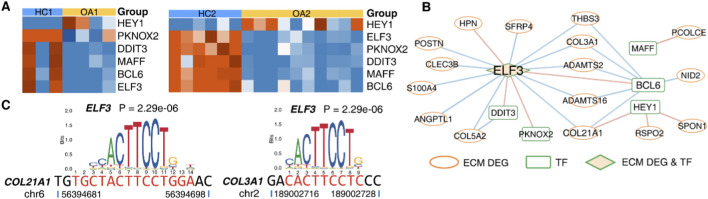
Transcriptional Regulatory Network of TFs and ECM-related DEGs. **(A)** Heatmap of DE TFs between OA and HC groups. **(B)** Co-expression network between DE TFs and ECM-related DEGs. Edge colors represent correlation types: red = positive, blue = negative. **(C)** Computational prediction of *ELF3* transcription factor binding sites within the promoter regions of *COL21A1* and *COL3A1* genes.

To uncover the regulatory mechanisms of the expression of ECM-related genes in OA, we analyzed the differentially expressed transcription factors (DE TFs) that were commonly identified in the OA and HC groups across the two datasets. Heatmaps were used to display their expression levels in each sample. It was observed that, compared to the HC group, the transcription factor genes *PKNOX2*, *DDIT3*, *MAFF*, *BCL6*, and *ELF3* were significantly downregulated in OA, while only the transcription factor *HEY1* was upregulated in OA (Figure 3A). By conducting correlation analyses between DE TFs and ECM-related DEGs, we constructed a co-expression network of DE TFs and ECM-related DEGs (Figure 3B). The transcription factor gene *ELF3* occupies a central position, interacting with multiple ECM-related DEGs (such as *S100A4*, *ANGPTL1*, *COL5A2*, *COL21A1*, etc.), and it is also regulated by transcription factors *BCL6*, *DDIT3*, and *PKNOX2*. Subsequently, we employed bioinformatics prediction to analyze the potential binding sites of *ELF3* in the promoter regions of *COL21A1* and *COL3A1*. Motif analysis revealed a potential *ELF3* binding site in the promoter region of *COL21A1*, with the sequence 5′-CAATTCCTC-3’; a potential *ELF3* binding site was also identified in the promoter region of *COL3A1*, with the sequence 5′-CACTTCCTC-3’ (Figure 3C).

### 3.3 Analysis of ECM-related splicing events in OA

**FIGURE 4:**
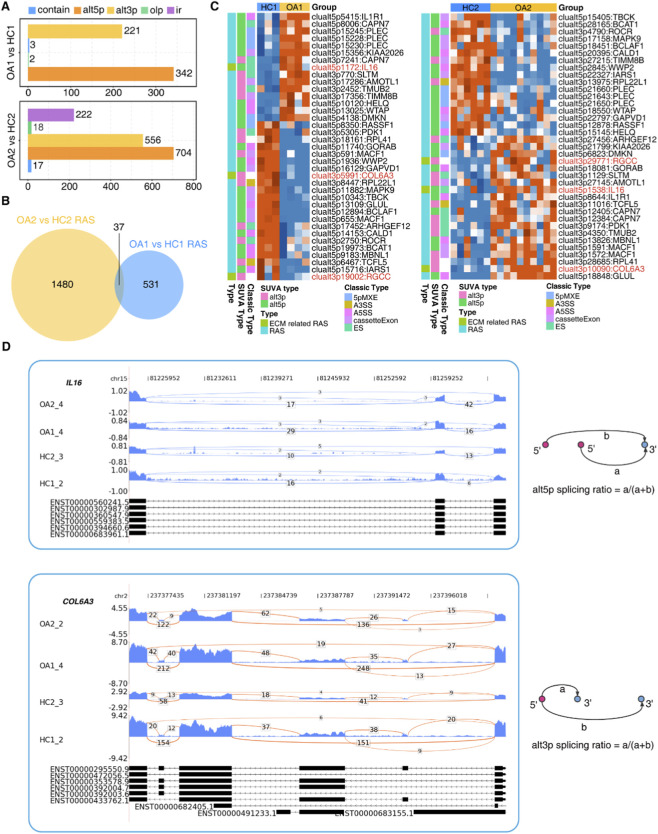
ECM-RAS Events. **(A)** Bar plot of SUVA-identified RAS types. **(B)** Venn diagram showing overlapped RAS events between datasets (n = 37). **(C)** Heatmap of splicing ratios for overlapped RAS events. ECM-related events highlighted in red. **(D)** Sogen plots depicting AS patterns of *IL16* and *COL6A3* genes.

To further explore the role of AS in the pathogenesis of OA, we utilized the SUVA to identify AS events in the two datasets. Initially, we sought to identify RAS events between HC and OA groups. Altogether, 568 and 1517 RAS events were identified by SUVA in Dataset 1 and Dataset 2, respectively, with alternative 5′splice sites (alt5p) and alternative 3′splice sites (alt3p) being the predominant types (Figure 4A). Corresponding to classical splicing events, they were predominantly cassette exons and alternative 5′splice site selection (A5SS) (Supplementary Figure S2A). The GO-BP analysis of these regulated alternative splicing genes (RASGs) showed that they were enriched in processes related to RNA splicing and cell structure (Supplementary Figure S2B). Further overlap analysis revealed that 37 RAS events were present in both datasets (Figure 4B; Supplementary Table S2). This indicates that these AS events may have a certain degree of conservation in the regulation of OA pathogenesis. The heatmap displays the splicing ratios of these 37 RAS events in each sample from the two datasets and annotates their types. Subsequently, 3 ECM-RAS events were identified, involving RASGs *COL6A3*, *IL16*, and *RGCC* (Figure 4C). In OA, *IL16* tends to utilize the proximal 5′splice site, while *COL6A3* and *RGCC* preferentially select the distal 3′splice site (Figure 4D; Supplementary Figure S2C).

### 3.4 Identification of RBPs regulating ECM-related splicing events in OA

**FIGURE 5:**
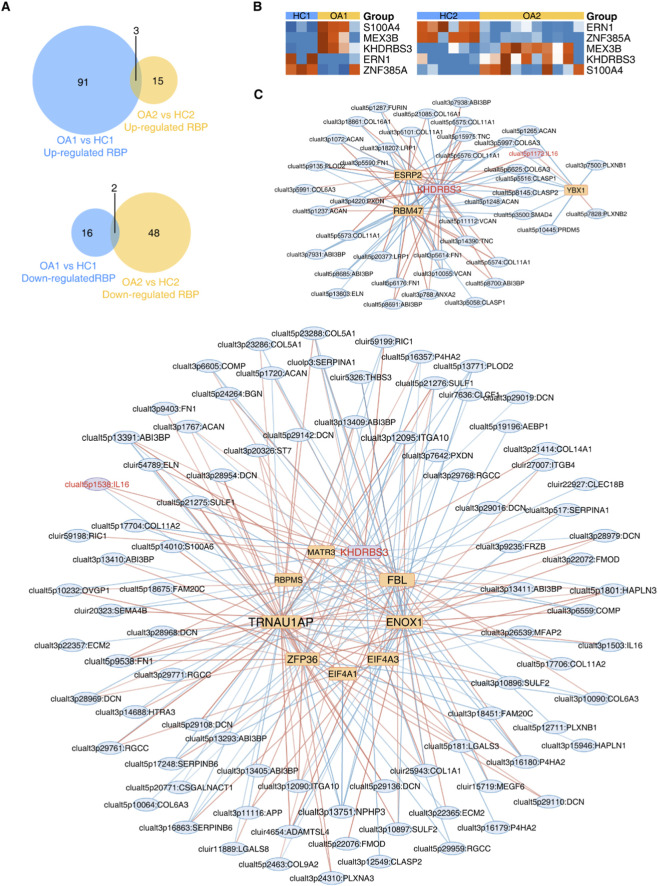
Regulatory Network of RBPs and ECM-related Splicing Events. **(A)** Venn diagram of overlapped RBPs between datasets. **(B)** Heatmap showing expression patterns of overlapped RBPs. **(C)** Co-regulation network between DE RBPs (rounded rectangle) and ECM-RAS events (ellipse), with red/blue edges indicating positive/negative correlations.

AS, as a crucial post-transcriptional regulatory mechanism, is significantly influenced by RBPs that determine the final form of mRNA and the resulting protein products. We conducted predictive analysis of the upstream RBPs involved in ECM-related splicing events in OA. By intersecting the two datasets, a total of 5 differentially expressed RBPs (DE RBPs) were identified (Figure 5A). Compared with the HC group, the expression of *S100A4*, *MEX3B*, and *KHDRBS3* was upregulated in OA, while the expression of *ERN1* and *ZNF385A* was downregulated (Figure 5B). We further constructed a correlation network between DE RBPs and ECM-RAS events (Figure 5C). It was found that KHDRBS3 could regulate the AS of the ECM-related gene *IL16* in both datasets. This suggests that the RNA-binding protein KHDRBS3 may contribute to ECM homeostasis disruption and the pathogenesis of OA via regulating AS of *IL16*.

### 3.5 Analysis of key molecules in OA based on single-cell RNA-seq data

**FIGURE 6:**
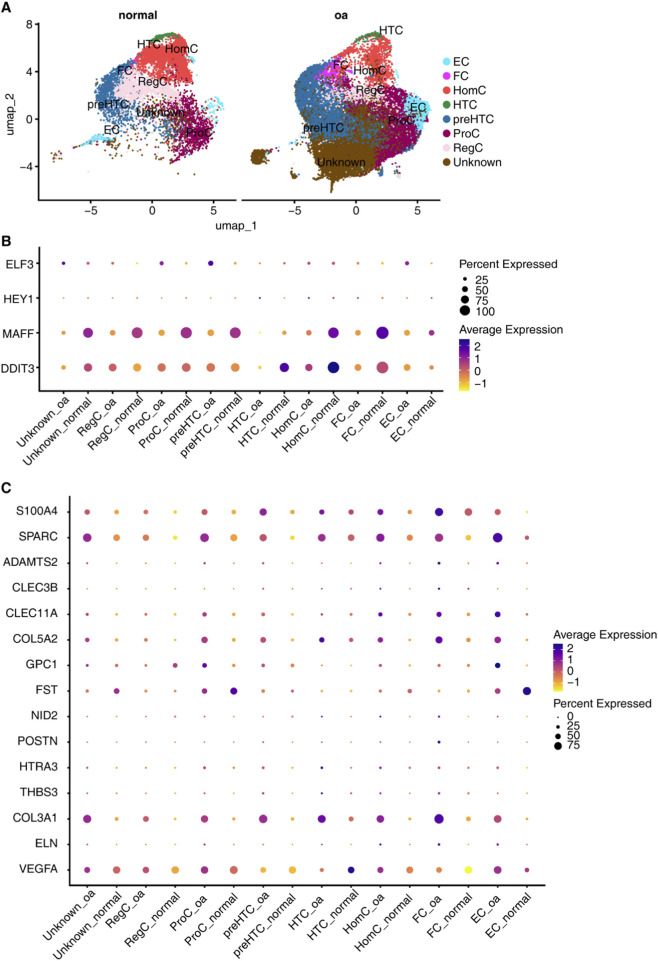
Single-cell Validation of Key Regulators. **(A)** UMAP projection of chondrocyte subtypes: Fibrochondrocytes (FC), Homeostatic (HomC), Hypertrophic (HTC), Pre-hypertrophic (preHTC), Proliferating (ProC), Regulatory (RegC), Effector (EC), and Unknown. **(B)** Dot plot showing conserved DE TFs across bulk and single-cell data. **(C)** Dot plot displaying overlapping ECM-related DEGs expression in chondrocyte subtypes.

To thoroughly and precisely uncover the pathogenesis of OA and the key molecular regulatory networks, we utilized scRNA-seq data (GSE169454) for validation, confirming the expression patterns of key genes and factors at the single-cell level. We first analyzed the cellular composition of the two groups of samples in the scRNA-seq dataset. After normalizing the single-cell transcriptome expression matrix, performing UMAP dimensionality reduction analysis based on principal components, and visualizing the results, we annotated the cell clusters based on known cell-specific markers. This process identified eight distinct cell clusters: fibrocartilage cells (FC), effector chondrocytes (EC), homeostatic chondrocytes (HomC), hypertrophic chondrocytes (HTC), pre-hypertrophic chondrocytes (preHTC), proliferating chondrocytes (ProC), regulatory chondrocytes (RegC), and an unknown type (Unknown) (Figure 6A). The UMAP analysis reveals a distinct distribution of chondrocytes in the low-dimensional space for OA compared to normal samples. Specifically, the proportions of HomC, HTC and RegC are reduced in OA, while the proportions of FC, EC, preHTC and ProC are increased (Figure 6A). These differences may be related to the pathogenesis of OA and the changes in chondrocyte states.

By analyzing the DE TFs between the two groups of samples in the scRNA-seq dataset and within each type of chondrocyte, and intersecting them with the DE TFs identified in the bulk RNA-seq dataset, we obtained four overlapping TF genes, specifically *ELF3*, *HEY1*, *MAFF*, and *DDIT3* (Figure 6B). Among them, *MAFF* and *DDIT3* showed consistent expression trends in both scRNA-seq and bulk RNA-seq data, with overall downregulation in OA. *DDIT3* was downregulated in the RegC, ProC, HTC, HomC, and FC types in OA, while it was upregulated in preHTC. The scRNA-seq data analysis revealed upregulation of *ELF3* in preHTC cells in OA, while bulk RNA-seq data show that the overall expression of *ELF3* is downregulated in OA patients (Supplementary Figure S3A–C). This apparent inconsistency is primarily driven by cell-type-specific expression patterns and variations in chondrocyte subtype proportions, rather than disease-stage heterogeneity or technical bias. Bulk RNA-seq reflects the average expression of genes across all cell types in cartilage tissue, so the overall downregulation signal likely arises from the combined effect of gene expression changes in the majority of chondrocyte subtypes; in contrast, single-cell RNA-seq captures subtype-specific expression, thus identifying the unique upregulation of ELF3/DDIT3 in preHTC. This difference highlights the advantage of single-cell resolution in dissecting gene expression heterogeneity that is masked in bulk analysis. This demonstrates that single-cell sequencing data can more precisely localize gene expression trends in different cell types.

Similarly, by performing overlap analysis between ECM-related genes identified in the scRNA-seq dataset and those in the bulk RNA-seq dataset, a total of 15 overlapping ECM-related genes were obtained. Among them, genes such as *S100A4*, *SPARC*, *COL5A2*, and *COL3A1* showed certain expression differences in various types of chondrocytes between the OA and normal groups, with upregulation in OA (Figure 6C), consistent with the bulk RNA-seq data.

In conjunction with the co-expression network of DE TFs and ECM-related genes, we speculate that the transcription factor ELF3 may specifically regulate the transcription of ECM-related genes such as *COL3A1*, *COL5A2*, and *S100A4* in preHTC, while the transcription factor DDIT3 may regulate the transcription of *COL5A2*, together mediating the pathogenesis of OA.

## 4 Discussion

This study elucidates the dynamic changes in the ECM during the progression of OA and explores the underlying complex regulatory networks, with a focus on the roles of transcriptional regulation and AS—a dual regulatory perspective rarely integrated in prior OA research. By analyzing two sets of OA transcriptome data, it was found that the DEGs were enriched in ECM-related pathways. We constructed a regulatory network of DE TFs-ECM-related DEGs, highlighting the potential regulatory roles of transcription factors ELF3 and DDIT3 in modulating the expression of ECM-related genes such as *COL3A1*, *COL5A2*, and *S100A4*. Notably, unlike previous studies that only reported ELF3’s general pro-degradative effect in articular chondrocytes (Otero et al., 2012; Zhang et al., 2024b; Otero et al., 2017), our scRNA-seq data further revealed that ELF3 is specifically upregulated in preHTC—linking its regulation of COL3A1/COL5A2/S100A4 to the preHTC-to-HTC differentiation axis, a process central to cartilage mineralization and OA progression. Additionally, our analysis of AS events identified KHDRBS3 as a regulator of the AS of the ECM-related gene IL16. While KHDRBS3 is known to regulate AS in tumors, its role in OA cartilage has never been reported previously. This fills a critical gap in understanding how RBPs mediate post-transcriptional regulation of ECM-related inflammation in OA.

Gene expression analysis demonstrated significant differences in the expression of ECM-related genes between OA and HC groups. The abnormal fluctuations in ECM-related gene expression may drive metabolic disturbances in chondrocytes and the abnormal activation of matrix-degrading enzymes (Hering, 1999; Kim et al., 2024; Arra and Abu-Amer, 2023). The continuous disruption of ECM homeostasis leads to the destruction of cartilage integrity and the loss of mechanical buffering functions (Choi et al., 2023; Chatterjee et al., 2022), confirming that ECM disruption and imbalance are key factors in OA progression. In the analysis of TFs, ELF3, an epithelial-specific member of the ETS transcription factor family, is involved in regulating the remodeling of the ECM (Otero et al., 2012; Zhang et al., 2024b; Otero et al., 2017). It has been reported that the expression of *ELF3* is upregulated in OA cartilage (Otero et al., 2017; Wondimu et al., 2018), which is consistent with our scRNA-seq data analysis results—but our single-cell data further clarifies that this upregulation is restricted to preHTC, rather than a global change across all chondrocyte subtypes. HEY1 acts as a negative regulator of osteoclast differentiation and plays a key role in bone metabolism (Rutkowski et al., 2016; Jin et al., 2022). DDIT3 is associated with endoplasmic reticulum stress, autophagy and inflammatory responses in OA (Yu et al., 2022; Huang et al., 2024; Yan et al., 2024). *DDIT3* has been identified as one of the definitive marker genes for HomC, and the expression of HomC in OA cartilage is significantly lower than that in healthy cartilage (Huang et al., 2025), which is consistent with our analysis results. BCL6 can alleviate IL-1β-induced OA progression by enhancing the transcription of *GPR61* (Zeng et al., 2025). A previous study has shown that the percentage of follicular helper T cells in patients with OA is relatively high. Follicular helper T cells induce B cells to produce immunoglobulins and express various genes (such as *BCL6*), and the expression of follicular helper T cells is positively correlated with the disease activity of OA (Chen et al., 2023). MAFF, a key transcription factor related to inflammation and lipid metabolism, is significantly downregulated in OA (Cao et al., 2022), although its specific mechanisms remain unclear. PKNOX2 is involved in cell growth, differentiation, and apoptosis (Li et al., 2023b), but no studies have yet linked it to OA.

Phenotypic changes in chondrocytes are a key factor leading to OA-related cartilage destruction. Using the scRNA-seq dataset GSE169454, we identified various chondrocyte subtypes that exhibit distinct and differential functional characteristics during the OA pathological process. Among them, FCs possess strong immunogenicity, not only highly expressing genes associated with poor outcomes but also having the ability to promote angiogenesis; these properties may collectively drive OA progression (Hu et al., 2022). The transition from normal cartilage (predominantly hyaline cartilage) to late-stage OA cartilage (dominated by FCs) further indicates the decline in normal cartilage function and the shift toward an abnormally proliferative fibrotic phenotype during OA progression (Pan et al., 2024). ECs are in the early stage of chondrogenic differentiation, with core functions of exerting immune effects and inducing tissue inflammation. They exhibit a significant role during OA progression and may be involved in processes such as disease development, tissue repair, or biological adaptation; their functional regulation tends to be associated with the TCA cycle, glycolysis, as well as lipid and amino acid metabolism (Hu et al., 2022; Chen et al., 2024). HomCs are highly similar to classical chondrocytes, remaining in a quiescent and fully differentiated state. They are the main contributors to ECM deposition, and due to their enrichment in circadian rhythm-regulating genes, they may play a key regulatory role in the circadian clock system of OA cartilage (Gan et al., 2021). PreHTCs have the ability to initiate and regulate the process of hypertrophic differentiation, serving as an important link between normal chondrocyte differentiation and pathological hypertrophy (Ji et al., 2019). HTCs are morphologically characterized by large size and absence of cell division, and they can regulate the mineralization of the surrounding matrix. Their presence further attracts blood vessels and osteocytes to invade cartilage tissue; subsequent cell apoptosis and calcium deposition ultimately accelerate the pathological progression of human OA (Ji et al., 2019). ProCs are flat and columnar, mainly distributed in the proliferative zone of the growth plate, with spatial differences in function: the upper-layer ProCs have the potential to inhibit hypertrophic differentiation, while the lower-layer ProCs (adjacent to preHTCs and HTCs) promote hypertrophic differentiation (Ji et al., 2019). RegCs are widely distributed throughout the entire process of chondrogenic differentiation, highly expressing genes related to immune responses, inflammatory responses, and responses to endogenous stimuli. Abnormal inflammatory responses can disrupt the activity of RegCs, thereby impairing cartilage tissue homeostasis and leading to damage to cartilage structure and joint function (Ji et al., 2019).

According to the DE TFs-ECM-related DEGs regulatory network and the verification analysis of scRNA-seq, we speculate that ELF3 and DDIT3 may influence the ECM of chondrocytes in preHTC by regulating the transcription of *COL3A1*, *COL5A2*, and *S100A4*. This regulatory role is particularly relevant to OA pathogenesis, as preHTC and their differentiation into HTC are central to cartilage degeneration: HTC are morphologically distinct, non-proliferative chondrocytes that regulate matrix mineralization, and their presence promotes vascular and osteoclast invasion into cartilage, followed by chondrocyte apoptosis and calcium deposition—ultimately accelerating OA progression (Ji et al., 2019). PreHTC, as the precursors of HTC, directly control the initiation of hypertrophic differentiation (Ji et al., 2019). In OA, articular cartilage responds to injury by shifting toward a degradative, hypertrophic-like state, characterized by abnormal ECM synthesis and elevated collagenase activity, and chondrocyte hypertrophy itself induces chondrocyte damage (Singh et al., 2019). The three target genes of ELF3 and DDIT3 further underscore their functional relevance to ECM homeostasis. *COL3A1* is highly expressed in fibrocartilage and pre-hypertrophic chondrocytes, which is consistent with the results of our analysis, and it maintains collagen homeostasis and contributes to ECM flexibility and elasticity (Li et al., 2022; Zhang et al., 2021). COL5A2 indispensable for collagen fibril assembly, a process critical for maintaining the structural integrity of the ECM (Sh et al., 2024). S100A4, a well-characterized marker of fibroblasts (Simonds et al., 2022), can promote chondrocyte hypertrophy by stimulating MMP13 signaling and participates in ECM remodeling (Yammani et al., 2009). Collectively, these genes are essential for preserving ECM structure, regulating ECM function, and mediating interactions between chondrocytes and their surrounding matrix. However, their expression patterns and functional roles in specific chondrocyte subtypes (e.g., preHTC, HTC) remain incompletely characterized in existing literature. Integrating our findings, we propose that the transcription of COL3A1, COL5A2, and S100A4 is tightly regulated by ELF3 and DDIT3. Dysregulation of these TFs (e.g., abnormal upregulation of ELF3 in preHTC or downregulation of DDIT3 in HomC) would disrupt the expression of these ECM-related genes, altering the ECM microenvironment surrounding chondrocytes. This perturbation, in turn, impairs chondrocyte function and phenotypic stability, creating a feedforward loop that drives cartilage degeneration and OA development. Based on these analyses, we speculate that ELF3 holds potential as therapeutic targets for OA, but attention must be paid to the potential risks of non-specific regulation. As an epithelium-specific member of the ETS transcription factor family, ELF3 functions are not limited to cartilage tissue (Otero et al., 2017); if ELF3 inhibition for OA fails to achieve cartilage specificity, its normal roles in other epithelial tissues or development-related pathways may be disrupted, thereby triggering potential functional abnormalities.

In terms of AS, the RAS events identified in our study were significantly enriched in RNA splicing and cell structure-related processes, which are closely related to the pathogenesis of OA. These abnormalities may lead to the dysfunction of chondrocytes and the inability to maintain the homeostasis of the ECM. Abnormalities in cell structure-related processes can also affect the morphology and function of chondrocytes, weaken their protective effect on the joints, and promote the development of OA. We identified three ECM-related genes with specific splicing alterations in OA. Among them, COL6A3 may be involved in the regulation of the structural composition and mechanical properties of cartilage (Bloks et al., 2024). Variations in COL6A3 can affect chondrocyte phenotypes and the expression of ECM-related genes in OA (Bloks et al., 2024). IL16 is a pro-inflammatory cytokine that regulates OA progression as a direct target of Novel-miR-81 (Luo et al., 2024). RGCC is a cell cycle regulator (Guo et al., 2021), and its relationship with OA remains unclear. By constructing an RBPs-ECM-RAS network, we further identified KHDRBS3 as a potential regulator of the AS of the ECM-related gene *IL16*. Regarding KHDRBS3, it is a member of the STAR family and is primarily involved in regulating RNA alternative splicing, export, and stability, and a large number of studies have shown that it affects tumorigenesis by regulating the AS of mRNA (Wu et al., 2019; Zhao et al., 2023). According to our analytical results, we hypothesize that KHDRBS3 may promote *IL16*’s utilization of the proximal 5′splice site—which typically generates an isoform with a truncated or altered N-terminal region. This (truncation or alteration) can affect protein localization, dimerization, or binding to downstream receptors (Song et al., 2023)—and may consequently produce a truncated, more active IL16 isoform. Such an isoform could then amplify IL16-mediated inflammatory signaling and disrupt ECM homeostasis. While experimental verification is still needed, KHDRBS3 could thus be speculated to have potential as an OA therapeutic target—its ability to specifically regulate *IL16* alternative splicing (a pro-inflammatory factor in OA) may allow targeted modulation of OA-related inflammation without fully ablating IL16’s physiological functions, though risks may include unintended effects on the splicing of other genes due to its pleiotropic roles in RNA metabolism.

In conclusion, our study systematically explores the transcriptional and AS regulatory mechanisms related to ECM in OA through the integrated analysis of bulk and single-cell transcriptome data—this is the first study in OA to combine these two omics layers at single-cell resolution to dissect coordinated ECM regulatory networks. We found significant differences in the overall gene expression patterns between OA and normal samples, identified multiple DEGs, DE TFs, and RAS events, constructed relevant regulatory networks, and validated the expression patterns of key molecules at the single-cell level, providing a multidimensional perspective and rich data support for a deeper understanding of the pathogenesis of OA.

However, this study also has certain limitations. In terms of data, although we integrated multiple public datasets, these datasets may be influenced by factors such as sample origin and experimental methods, potentially introducing heterogeneity that could interfere with the accuracy and reliability of the analysis results. The limited sample size in the datasets may also fail to comprehensively cover all pathological changes and individual variations in the complex disease of OA, restricting the universality and representativeness of the findings. In terms of the depth of mechanistic research, although we identified key TFs, RBPs, and related genes, our understanding of their specific molecular mechanisms and signaling pathways in OA pathogenesis remains limited. For example, how these molecules interact with each other, how they affect chondrocyte proliferation, differentiation, and apoptosis, and how they interact with other cell types to collectively regulate OA development all require further in-depth investigation. Additionally, all conclusions in this study are derived from bioinformatic analysis of public transcriptome data, and lack experimental validation in in vitro or *in vivo* models. To address this, follow-up experiments could be designed as follows: (1) *In vitro*, use siRNA or overexpression plasmids to knock down or overexpress *ELF3*, *DDIT3*, and *KHDRBS3* in primary human articular chondrocytes induced by IL-1β (to mimic OA-like pathological conditions), then detect changes in the expression of downstream target genes (*COL3A1*, *COL5A2*, *S100A4*) via qPCR/Western blot and changes in *IL16* AS patterns via RT-PCR; (2) *In vivo*, construct OA mouse models (e.g., destabilization of the medial meniscus, DMM); and perform chondrocyte-specific knockout of *ELF3* or *KHDRBS3*, then evaluate cartilage degeneration via histological staining (Safranin O-Fast Green) and assess changes in ECM-related gene expression and AS events in cartilage tissue to verify the functional roles of these key molecules in OA progression.

## Data Availability

The original contributions presented in the study are included in the article/Supplementary Material, further inquiries can be directed to the corresponding author.
