# Supplementary material for: A transcriptomic atlas at bulk and single-cell levels identifies novel transcriptional and splicing regulators of ECM homeostasis in osteoarthritis
Source: Front Genet. 2025 Dec 16;16:1690319. doi: 10.3389/fgene.2025.1690319 (PMC12748231; doi:10.3389/fgene.2025.1690319)
Supplement: Supplementary file 2 [file DataSheet1.docx]

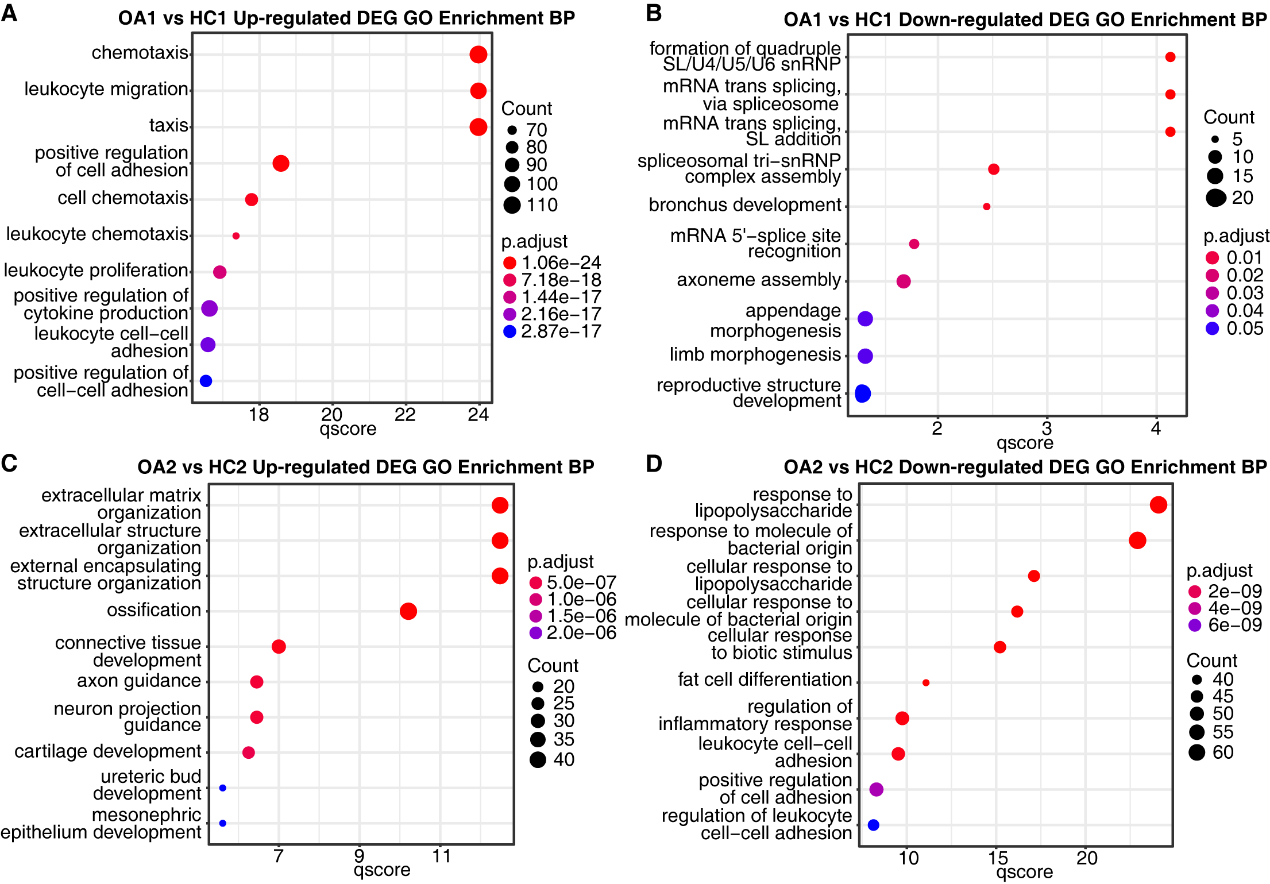


Figure S1. DEG GO Enrichment.

**(A-D).** GO enrichment analysis of up-/down-regulated DEGs in (A,B) GSE168505 and (C,D) GSE111357 datasets.


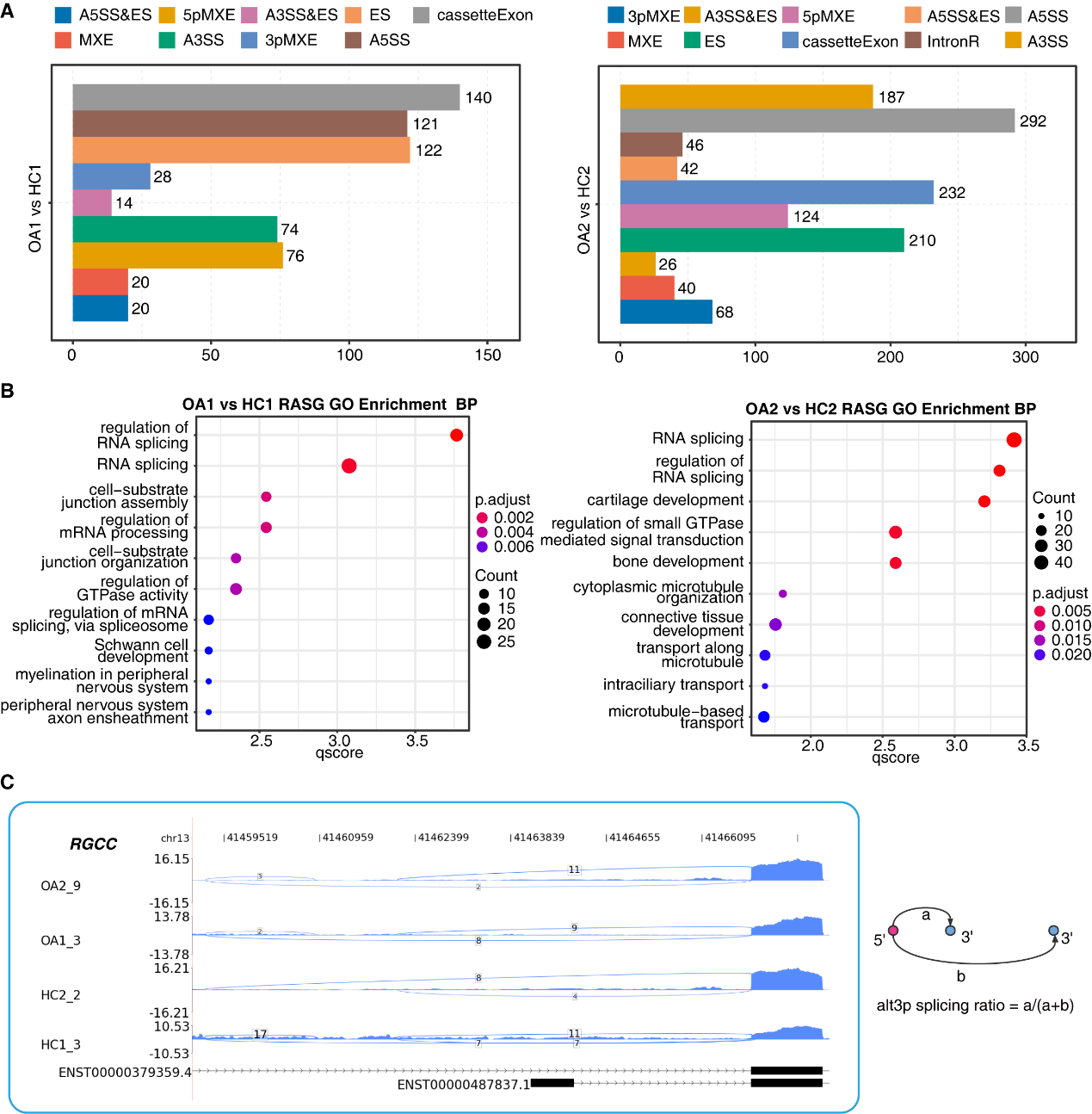


Figure S2. Additional Splicing Analyse

A. Classic alternative splicing types distribution.

B. GO enrichment analysis of RASGs.

C. Sogen plot of *RGCC* splicing events.


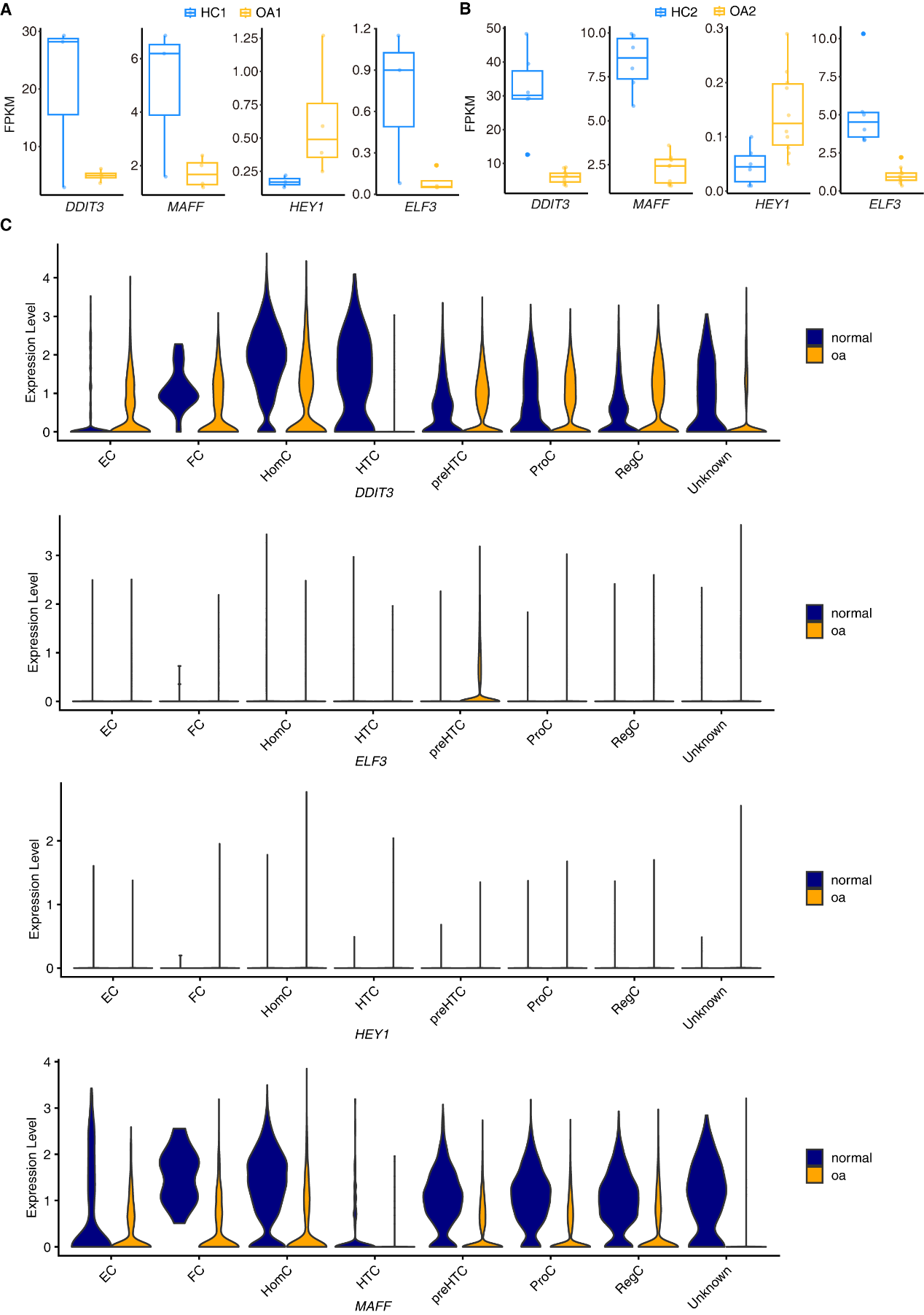


Figure S3. Consistency of Transcription Factor Expression Profiles Between Bulk RNA Sequencing and Single-Cell RNA Sequencing.

(A, B) Box plots showing detailed expression patterns of transcription factors (TFs) across experimental groups.

C. Violin plot depicting TF expression variations in different cell types among groups.
